# Medical Society

**Published:** 1897-10-30

**Authors:** 


### MEDICAL SOCIETY.

### Some Observations on the Localising Factors in Rheumatism and Chorea.

Dr. Churton, of Leeds, read a paper on this subject. Nearly 600 cases were tabulated, the state of health before the attack, the assigned causation and the joints first affected, the occurrence of acetonuria, indicanuria, and constipation being noted. The previous health was decidedly not good in a great majority of the cases. The causes assigned were chiefly wettings and injuries; in some of the cases, in which no causation could be assigned, indicanuria was found. It was constantly observed that the joints of those limbs which were chilled or injured were the first to suffer. There was no positive exception to this rule, although in a few cases, probably owing to imperfect information, there were apparent exceptions. The "toxine + local injury" theory of rheumatism had been held by Sir Dyce Duckworth and others. The toxine might perhaps be glycocine (Latham); if so, the micro-organism would not be found where it has been sought—in the blood, joints, &c. Moreover, disorder of the digestive epithelia and muscles may well be caused by shock to the brain; hence the rheumatism toxine may hereafter be found to be caused indirectly, and the localisation of its effects directly, by the same shock; or the latter by either simultaneous or subsequent chills or injuries. In 150 cases of chorea tabulated it was, with respect to most of them, impossible to reply to the questions—how is the connection of the chorea and rheumatism shown? and even when the patient was admittedly rheumatic, why was the action of the toxine diverted to the brain? A few of the cases showed the localising power of shock in rheumatic patients, thus bringing chorea more sharply into line with arthritis in this respect. Among the cases mentioned was one of a girl, aged 18, the child of an alcoholic father; overwork in an insanitary house was followed by chorea and arthritis. She became sullen and apparently insane, but recovered under the free administration of port wine after failure of other means, including inhalation of chloroform with morphia at the same time hypodermically. In the cases tabulated there was no instance of chorea primarily caused by chill, and no case of arthritis primarily caused by fright.

The paper was very fully discussed, and Dr. Churton in reply mentioned a case in which right brachial monochorea followed a blow on the left side of the head. Dr. Mackenzie's reference to anæmia as a post-rheumatic symptom suggested that anæmia might be one of the signs of pre-existing rheumatic poisoning in choreic children.

